# Arthroscopic reduction and fixation of coronoid fractures with an exchange rod—a new technique

**DOI:** 10.1186/s13018-016-0505-8

**Published:** 2017-01-18

**Authors:** Kan Ouyang, Daping Wang, Wei Lu, Jianyi Xiong, Jian Xu, Liangquan Peng, Haifeng Liu, Hao Li, Wenzhe Feng

**Affiliations:** 1 Department of Sports Medicine, Shenzhen Second People’s Hospital, 1st Affiliated hospital of Shenzhen University, Shenzhen, 518035 Guangdong Province China; 2 Department of Orthopaedics, Shenzhen Second People’s Hospital, 1st Affiliated hospital of Shenzhen University, Shenzhen, Guangdong Province China

**Keywords:** Arthroscopy, Bone nails, Fracture fixation, Internal, Ulna fractures

## Abstract

**Background:**

The ulnar coronoid process plays a central role in maintaining elbow stability. Some of its fractures were often combined with injury of bone and ligament. Arthroscopy enables perfect visualization to allow anatomical repair.

**Methods:**

From January 2012 to December 2013, six patients (four males, two females) with a mean age of 26.6 years were treated. The left and right ulnas were involved in two and four patients, respectively. All patients suffered from ipsilateral subluxation of the elbow without associated radial fracture. According to the Regan and Morrey fracture classification and O’Driscoll’s classification, two and four patients were classified as type I and type II and as having tip fracture (O’Driscoll type I) and anteromedial fracture (O’Driscoll type II), respectively. Exchange rod technology via the elbow front center approach was used for reduction and fixation of fractures of the coronoid process of the ulna.

**Results:**

Intra- and postoperative X-ray examination showed that the fractures were satisfactorily fixed and that the screw and fracture line were vertical to each other. Follow-ups showed that the fractures had healed well, and the average elbow extension was −2° while the average flexion was 140°. No problems related to pronation or supination, elbow instability, or complications of blood vessels or nerves were reported. The elbows showed excellent results according to the Mayo Elbow Performance Score.

**Conclusions:**

Arthroscopy using an exchange rod can provide excellent visual exposure of the fractured joints, without the need for a large incision during the anatomical repair. Moreover, it protects the surrounding soft tissue, shows good stability of the components, and allows early rehabilitation exercises.

## Background

The ulnar coronoid process (CP) plays a central role in maintaining elbow stability [1–4]. Its fracture is not uncommon; it seldom occurs in isolation and is often accompanied by other fractures and/or ligament damage, consequently leading to elbow instability, which makes it a difficult fracture to handle [5]. According to Regan and Morrey classification [6], coronoid process fracture can be divided into three types including type I tip fracture, type II with fracture of 50% or less of height, and type III with fracture of more than 50% of height. Later, O’Driscoll had classified the coronoid process fracture into more subtypes [7]. Subtype I fracture was usually associated with posterior elbow dislocation injury, whereas subtype II and subtype III fractures were associated with varus subluxation.

At present, the consensus is to stabilize all fractures of the CP associated with elbow instability [8]. The goal of treatment is to obtain a stable, pain-free, and functional elbow. Treatment should be begun as early as possible and be associated with early rehabilitation and short lasting immobilization. It has been believed that only type III fractures require open reduction and internal fixation to improve elbow instability [9]. However, in recent years, several researchers observed that types I and II fractures also need treatment because of the combined injury of bone and ligament [8–11]. Therefore, prompt anatomical reduction is recommended. Different surgical techniques have been described: suture lasso, screws, plates, and tension band wiring with steel wire (*an original internal fixation technique by tension band wiring with steel wire in fractures of the coronoid process*). Fixation of the coronoid fragment again depends on location and size. Smaller fractures associated with the “terrible triad” or varus posteromedial instability can be stabilized by “lasso-type” sutures through proximal ulnar drill holes or suture anchors both incorporating the fragment’s capsular attachment. Larger fragments can be fixed by screws as necessary with cannulated or non-cannulated screws. Large anteromedial facet fractures can be secured with precontoured or T-plates in a buttress fashion [8, 12].

Almost all the surgical techniques noted above using open surgeries which typically require a fairly large incision and the small fracture fragment may slide into the posterior compartment of the elbow. However, the deep intra-articular location of the CP makes an approach by open surgery as well as reduction difficult. Arthroscopy can help obtain intra-articular control of fracture reduction which enables perfect visualization to allow anatomical repair. This study described six patients in whom the exchange rod arthroscopic technique was effectively used for the reduction and fixation of fractures of the coronoid process of the ulna.

## Methods

### Patients and fracture classifications

In total, six patients (four males and two females) with an average age of 26.6 (ranged from 19 to 34) years were recruited; two patients had a fracture of the left ulna while four patients had a fracture of the right ulna. All six patients had ipsilateral elbow subluxation and coronoid process fracture without radial fracture. According to the Regan and Morrey [6] fracture classification, two patients were classified as type I, and four patients were classified as type II. According to O’Driscoll’s [13] typing method, two patients were classified as having tip fracture (O’Driscoll type I), and four patients were classified as having anteromedial fracture (O’Driscoll type II). All fractures were consequences of indirect violence.

**Fig. 1:**
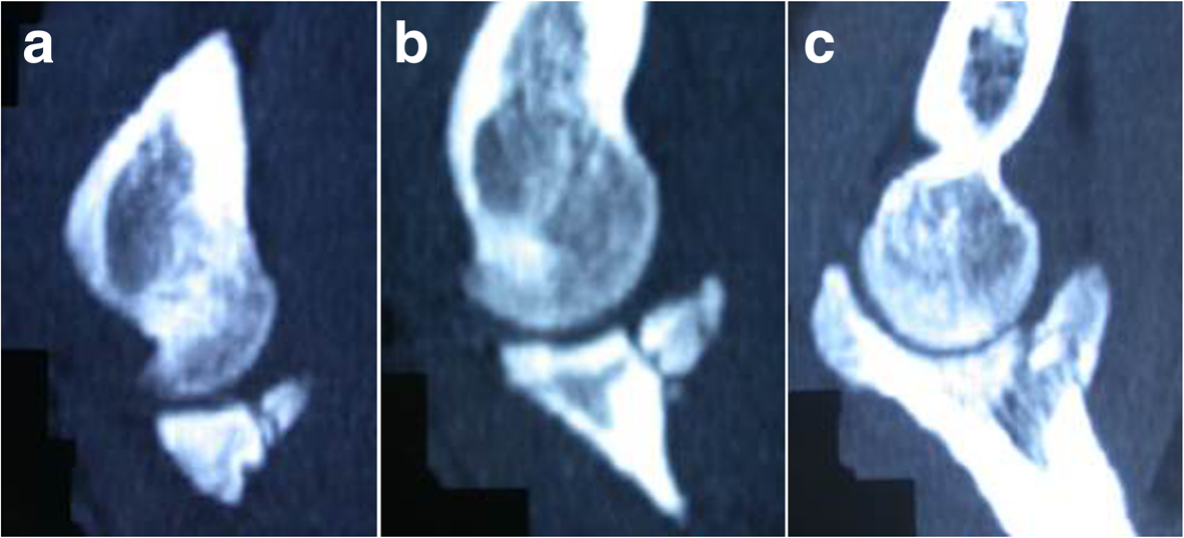
**a**–**c** CT scan of a 32-year-old male patient shows fracture of the ulnar coronoid process (Regan and Morrey type II)

All patients underwent preoperative X-ray examination, computed tomography (CT), and magnetic resonance (MR) imaging of their elbow for assessment (Fig. 1). In awake as well as anesthetized patients, humero-ulnar instability between 25° elbow extension and full extension was observed. CT and MR imaging aimed to check the degree of instability for posterolateral and posteromedial rotation. Further, MR imaging could assess the integrity of the medial or lateral collateral ligament. The instability always existed if there were obvious ligament tear and complex fractures observed from MRI or CT. However, not all these examinations can observe the positive signs regarding the instability. In our study, none of the patients got comminuted ulna fractures and were complicated with radial fractures in which arthroplasty, open reduction, or internal fixation of radial fractures were not needed.

### Surgical technique

**Fig. 2:**
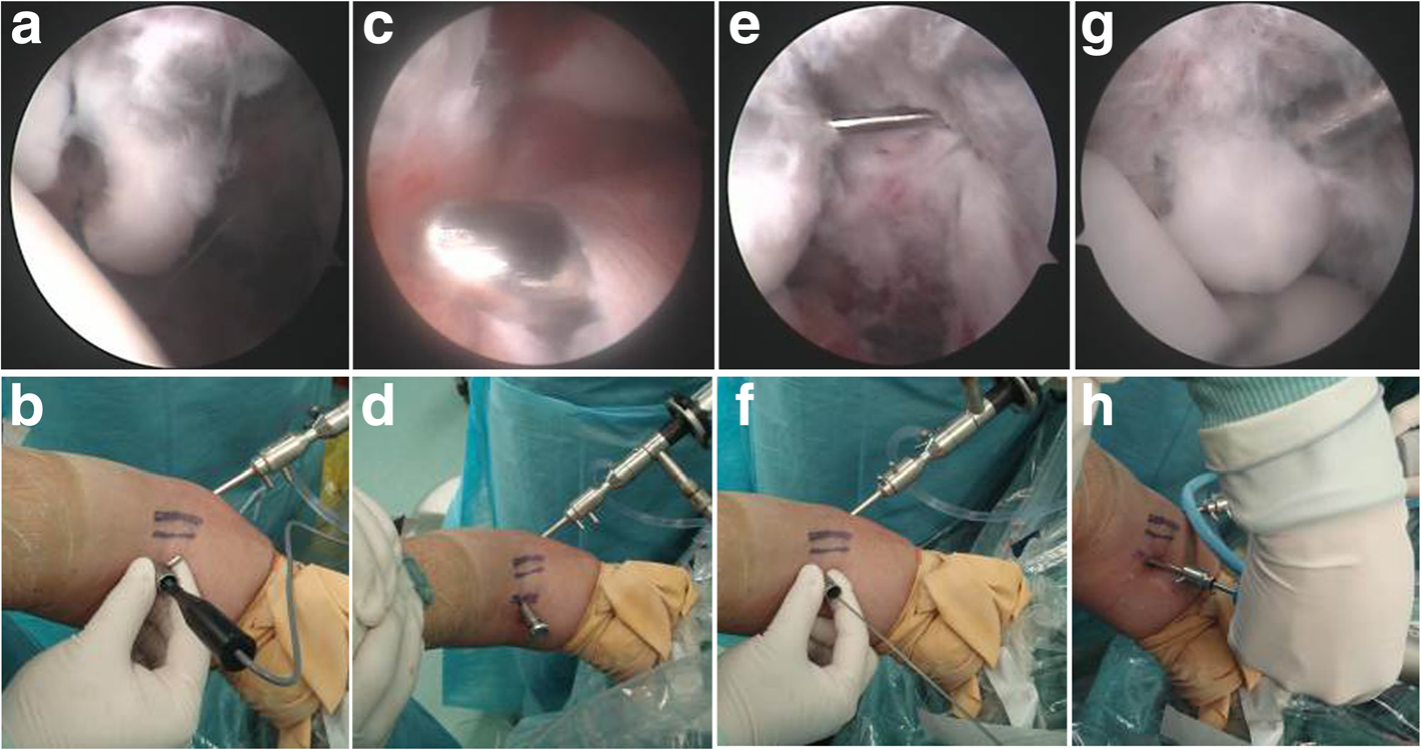
**a**–**h** Exchange rod arthroscopic techniques for the reduction and fixation of fracture of the ulnar coronoid process: clean fracture surface, fracture reduction (**a**, **b**); exchange rod technique for midline approach (**c**, **d**); Kirschner wire pierced vertical to the bone (**e**, **f**); and screwed into the hollow screw (**g**, **h**)

The patient was laid in a supine position on the operating table, with the upper limb, the elbow, and the upper limb flexed forward to 90° and the forearm flexed to nearly 30°. Accurate anesthetization of the arm was ensured. The entry points to the elbow were marked before inflating the tourniquet to 250 mmHg. The elbow joint was then checked according to the ISAKOS (The International Society of Arthroscopy, Knee Surgery and Orthopaedic Sports Medicine) classification [14]. Subsequently, the arthroscopy was introduced via the proximal, anteromedial, and lateral approaches, and the soft tissue around the fracture block was cleared, separating it from the outer tissues. The fracture site was then refreshed by removing any tissue that prevented a good reduction and ultimate fixation. The tourniquet was released. A 10-mm incision was made transverse to the surface of the bicep tendon, avoiding the cephalic vein by bluntly dissecting clear down to the bicep tendon surfaces. The lateral cutaneous nerve of the forearm was carefully protected and tracted laterally with the skin and subcutaneous tissue. The index finger was used to feel and separate the blood vessels, nerves, muscle, and other tissues. The fingertip was used to separate and gradually reach the anterior capsule of the elbow joint. At this point, the anterior capsule was pushed with the index finger and visualized through the arthroscope. With the index finger still in situ, a blunt exchange rod measuring about 3 mm in diameter was introduced along the pulp of the index finger, through the anterior capsule. Next, a cannula measuring 3.5 mm in diameter was introduced over the exchange rod. A Kirschner wire of 1.5 mm diameter was then passed through this cannula into the joint. With the fracture accurately reduced, the wire was inserted vertical to the posterior cortex of the bone fragment. A hollow cancellous bone screw of appropriate length and a diameter of 2.0 or 3.5 mm was selected and fixed into the bone, using the wire as a guide (Fig. 2). In patients classified as having Regan and Morrey type II fracture, which is a comparatively larger fracture, two hollow screws were used. After fixation, the extension and flexion of the joint was checked to assess the stability of fixation, particularly when it was under the valgus stress. Under arthroscopy, the external rotation shift and the width of the brachial, ulnar, or medial artery or the subluxation of the humeral bone and caput radii were tested. All surgeries were finished in 90 min, with the average bleeding amount no more than 20 ml.

### Postoperative treatment and efficacy evaluation

After treatment, the elbow was kept immobilized in a plaster for 2–3 days, followed by encouraging gentle active movements, avoiding violent massage to prevent the occurrence of myositis ossificans. The outpatients were regularly reviewed to assess the overall function of their elbow joint, based on the Mayo Elbow Performance Score (MEPS) scoring system [3], considering factors such as pain (45 points), range of motion (20 points), stability (10 points), daily function (25 points), and so on, with ≥90 points scored as A, 75–89 points scored as B, 60–74 points scored as C, and ≤60 points scored as D.

## Results

**Fig. 3:**
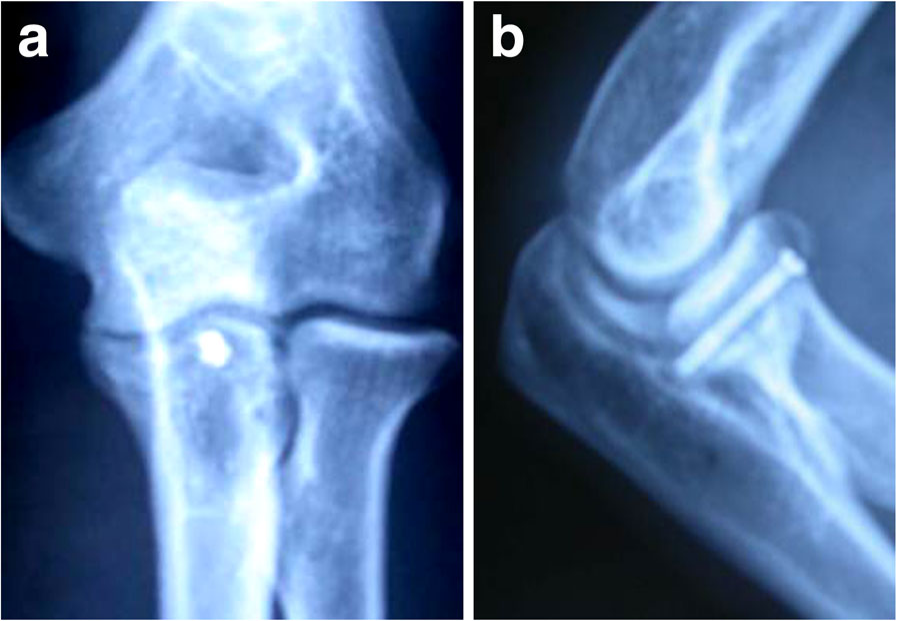
**a** X-ray of a 32-year-old male patient shows fracture of the ulnar coronoid process (Regan and Morrey type II). **b** Lateral X-ray 6 weeks after the treatment shows no displacement of the fracture

**Fig. 4:**
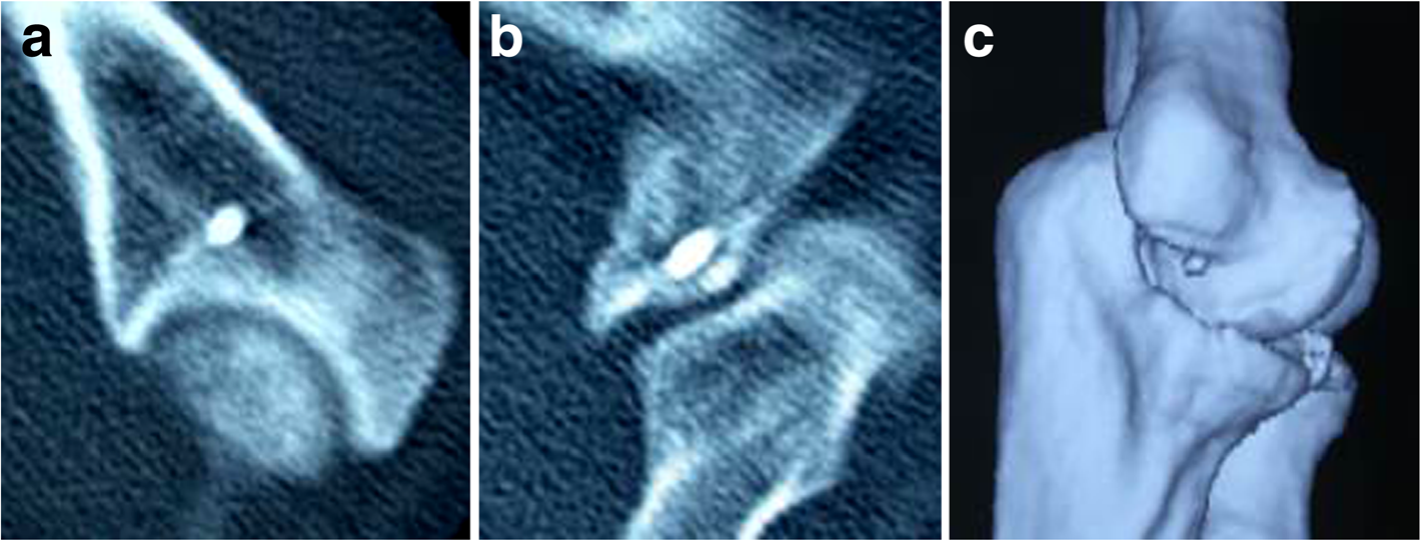
**a**–**c** Six weeks postoperative CT scans showed no fracture displacement

**Fig. 5:**
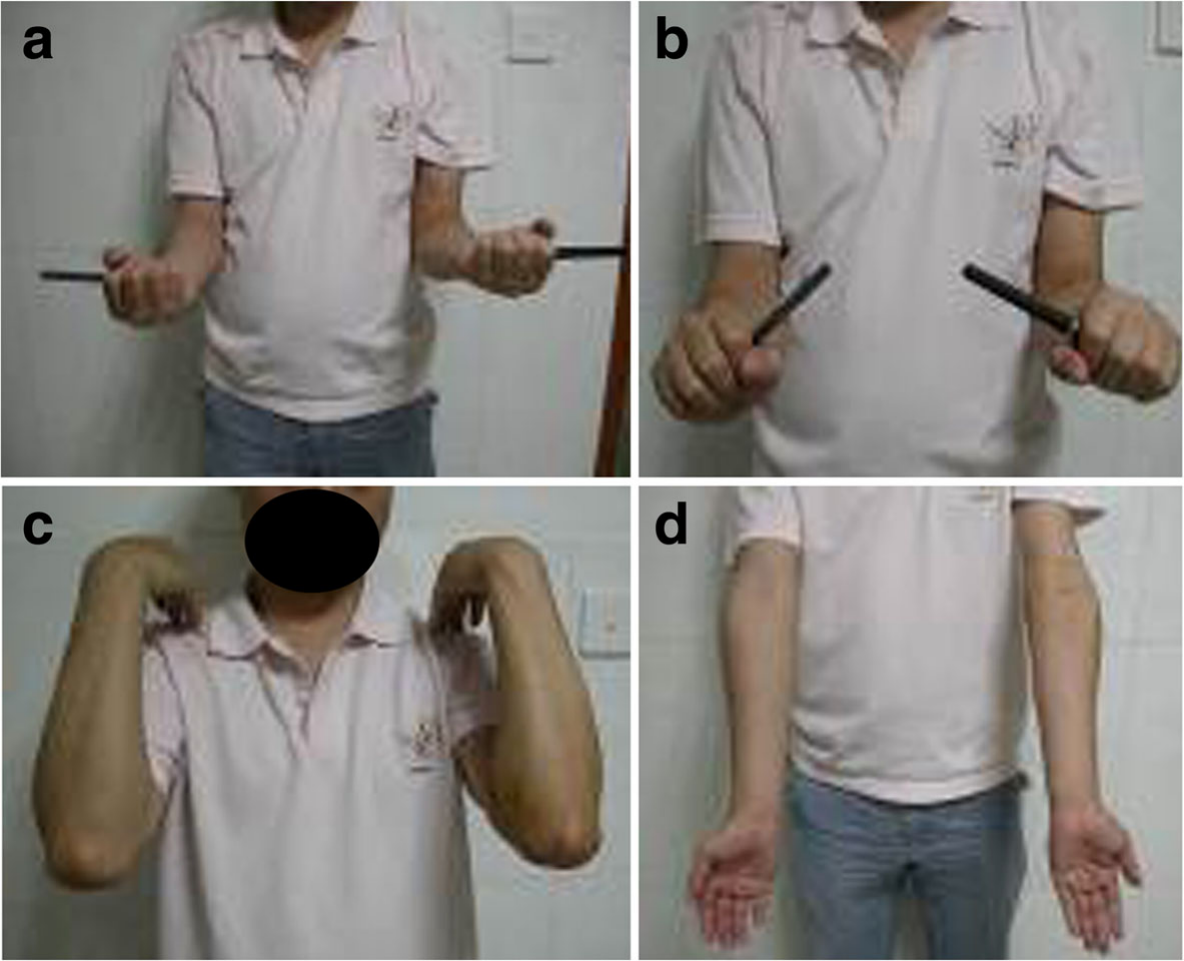
**a**–**d** Images of a 32-year-old male patient with fracture of the ulnar coronoid process (Regan and Morrey type II) 1 year after treatment show normal elbow pronation (**a**) and supination (**b**) and flexion (**c**) and extension (**d**)

X-ray examinations conducted at the time of surgery showed that all fractures were anatomically reduced. Five patients were followed up for an average of 11 (range, 7–24) months; one patient was lost to follow-up (Table 1). The fractures in all five patients had healed well. Lateral X-ray of the elbow and CT scans conducted 6 weeks after the treatment and at the end of the follow-up period showed no further displacement of fracture (Figs. 3 and 4). At the end of the follow-up, all patients were able to completely bend their elbow. Four patients could extend their elbow completely, while one patient could not fully extend his elbow, with a shortfall of 10°. The elbow extension in all five patients averaged −2° (range, −10° to 0°), while the average flexion was 140° (range, 135° to 145°). No problem related to pronation or supination or elbow instability was reported in any patient (Fig. 5). No blood vessel or nerve damage was observed during the 1-year follow-up period. All five patients showed an MEPS score of A.

**Table 1:** The postoperative condition of the patients during the follow-up

Patient no.	Postoperative X-ray	CT scans	Range of motion	Elbow stability	Blood/nerve damage	MEPS score
1	Good alignment	No displacement	−2° to 135°	Stable	None	A
2	Good alignment	No displacement	−4° to 135°	Stable	None	A
3	Good alignment	No displacement	0° to 145°	Stable	None	A
4	Good alignment	No displacement	−3° to 140°	Stable	None	A
5	Good alignment	No displacement	−1° to 145°	Stable	None	A

## Discussion

The ulnar coronoid process plays a central role in stabilizing the elbow joint [7, 13, 15–18]. Fracture of the coronoid process is not uncommon; it seldomly occurs in isolation and is often accompanied by other fractures or ligament damage in the area, leading to elbow instability. Regan and Morrey [6] classified fractures based on the fragment size, with type III fracture accounting for more than 50% of the coronoid process fractures. It is believed that these fractures generally require open reduction and internal fixation in order to avoid recurrent elbow instability.

Even though type I fractures can usually be treated non-surgically, the optimal treatment for type I coronoid process fractures remains controversial [19]. O’Driscoll [7, 13] and Doornberg and Ring [15] reported that the elbow joint instability may result from a small fracture, such as Regan and Morrey types I and II or O’Driscoll types I and II fractures. Such fractures may be more complex than previously imagined and, when associated with ulnar collateral ligament or radial collateral ligament damage, may consequently lead to elbow instability. As is known, type III fractures can cause severe elbow instability; moreover, based on the extent of the bone injury rather than ligament injury, the surgeons usually opt for the safer and more reliable open fixation [7, 15, 20]. However, the types I and II fractures may often be ignored by treatment which makes the outcome more difficult to predict than type III fractures. The ligament injuries associated with a small coronoid fracture may play a more important role in elbow instability than the fracture itself. In fact, when these kinds of fractures show elbow joint instability, internal fixation is preferred [17].

Congruent stability of the elbow joint and anatomical fracture healing remain the primary goals of treatment [6, 7, 15]. A variety of operations for open reduction, internal fixation, and capsular repair require a larger incision [6, 16, 17, 20]. In case of an intact radius, open reduction for small coronoid process fractures can be technically challenging, since it requires extensive exposure of the fracture site and may result in the dissociation of the attached residual anterior capsule [17, 21]. Moreover, it may hinder the blood supply of the fracture fragments. The combination of small fracture fragment comminution and soft tissue stripping may result in insufficient fixation and residual instability. Also, the damage to the integrity of the anterior capsule would cause losing the function as the stabilizing structure. Using arthroscopy can help obtain intra-articular control of fracture reduction which enables perfect visualization to prevent damage to the capsules and protect the blood supply.

**Fig. 6:**
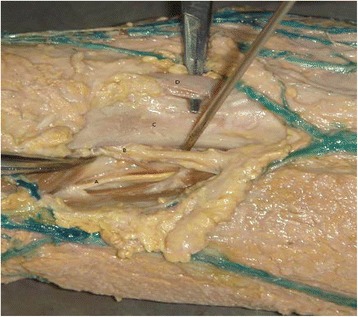
Anatomy of the elbow with a median approach using the Kirschner wire. *a* Radial nerve. *b* Lateral cutaneous nerve of the forearm. *c* Biceps tendon. *d* Elbow median neurovascular bundle (brachial artery, median nerve)

During the arthroscopy, the anatomic factors are of importance to consider. The anterior area of the elbow is rich in blood vessels and nerves; however, the area that is close to the outer flank of the biceps tendon is relatively safe (Fig. 6). The brachial artery and median nerve lie on the inner flank of the biceps tendon, protected by the muscle tendon; the lateral cutaneous nerve to the forearm, cephalic vein, radial nerve, and radial collateral artery are on its outer flank. The radial nerve and radial collateral artery lie between the brachial muscle and the brachioradialis muscle; however, in our approach, the incision is made well away from them, so they are not likely to be damaged. The lateral cutaneous nerves to the forearm and cephalic vein are comparatively shallow, so the incision is made in the skin alone, and dissected carefully. They are easily located and are pulled outside for protection. After blunt dissection with the index finger, the exchange rod technique is used to further reduce the risk of neurovascular injury. When the elbow joint is bent, the tension on the peripheral nerves, blood vessels, and tendons is reduced and the biceps tendon can be pulled slightly inside to expose the surface of the coronoid process. This enables the insertion of the screw vertical to the fracture line, which facilitates anatomical reduction and firm fixation. Some studies have reported that the Kirschner wire is introduced from the rear of the elbow, and, similarly, the screw is introduced into the hollow nail to fix the fractures [22]. This operation is more challenging, since it is difficult to accurately guide the Kirschner wire into its ideal position in the fracture fragment. However, arthroscopy can make accurate positioning of the wire and insertion of the screw easier [23].

In cases where the fracture fragment is too small for screw fixation, some studies have reported that the use of cerclage suture fixation has achieved good results [21]. However, our group of patients had sufficiently large fragments to allow screw fixation. One patient with Regan and Morrey type II fracture had a large fracture fragment and required two cannulated screws for fixation. All of our results showed fractures were healing well, and the elbows were in stable condition. Intra- and postoperative X-ray examination showed that the fractures were satisfactorily fixed and that the screw and fracture line were vertical to each other. Follow-ups showed that the fractures had healed well, and the average elbow extension was −2° while the average flexion was 140°. No problems related to pronation or supination, elbow instability, or complications of blood vessels or nerves were reported. The elbows showed excellent results according to the Mayo Elbow Performance Score.

On the basis of our preliminary study, we speculate that fractures of the coronoid process of the ulna that do not require obvious open reduction surgery can be treated by arthroscopic reduction and fixation by using the exchange rod technology, which provides excellent visualization and allows a good anatomical repair without extensive dissection of soft tissue. As with any study, our study had some limitations. The low incidence of this specific fracture pattern compelled us to study only a small number of cases. For these reasons, our results may not reflect the whole advantages of this arthroscopic technique. Meanwhile, more prospective research regarding the comparison of arthroscopic technique and other open surgical techniques need to be performed. Another limitation was the relatively short follow-up period. Because of this, the development of late complications such as posttraumatic arthritis or implant failure would not be assessed. Even though this was not a long-term follow-up study, our results showed arthroscopy with an exchange rod can be an efficient method in treating the coronoid process fractures.

## Conclusions

Arthroscopy using an exchange rod can provide excellent visual exposure of the fractured joints, without the need for a large incision during the anatomical repair. Moreover, it protects the surrounding soft tissue, shows good stability of the components, and allows early rehabilitation exercises.
